# Microbiological Quality of Ready-to-Eat Leafy Green Salads during Shelf-Life and Home-Refrigeration

**DOI:** 10.3390/foods9101421

**Published:** 2020-10-08

**Authors:** Alyexandra Arienzo, Lorenza Murgia, Ilaria Fraudentali, Valentina Gallo, Riccardo Angelini, Giovanni Antonini

**Affiliations:** 1 Department of Science, Roma Tre University, Viale Guglielmo Marconi 446, 00146 Rome, Italy; alyexandraarienzo@gmail.com (A.A.); ilaria.fraudentali@uniroma3.it (I.F.); riccardo.angelini@uniroma3.it (R.A.); 2 Interuniversity Consortium INBB National Institute of Biostructures and Biosystems, Viale delle Medaglie d’Oro, 305, 00136 Rome, Italy; lorenza.murgia@uniroma3.it (L.M.); valentina.w@inwind.it (V.G.)

**Keywords:** RTE salads, microbiological quality, shelf-life, MBS method

## Abstract

The market of ready-to-eat leafy green salads is experiencing a noticeable growth in Europe. Since they are intended to be consumed without additional treatments, these ready-to-eat products are associated with a high microbiological risk. The aim of this work was to evaluate the microbiological quality and safety of ready-to-eat leafy green salads sold in widespread supermarket chains in Lazio, Italy, on the packaging date during shelf-life and during home-refrigeration. The study also aimed to determine the differences between low-, medium-, and high-cost products. *Salmonella* spp. and *L. monocytogenes* were chosen as safety indicators as specified by European regulations while total aerobic mesophilic bacteria and *Escherichia coli* were chosen as quality indicators as suggested by national guidelines. Analyses were performed following the ISO standards and in parallel for the evaluation of total aerobic mesophilic bacteria, with an alternative colorimetric system, the Micro Biological Survey method, in order to propose a simple, affordable and accurate alternative for testing the microbiological quality of products, especially suitable for small and medium enterprises and on-site analyses. The study revealed high, unsatisfactory, total bacterial loads in all analyzed samples on the packaging date and expiry date and a very high prevalence of *Salmonella* spp. (67%) regardless of the selected varieties and cost categories; *L. monocytogenes* was not recovered aligning with the results obtained in other studies.

## 1. Introduction

Industrialized countries have recently faced an emerging demand for healthy and time-saving dietary solutions consistent with the modification in eating habits and the reduced time available for food preparation [1,2]. In particular, the consumption of ready-to-eat leafy green salads (RTESs) has experienced a noticeable increase in Europe and especially in Italy, where, following a 10% average annual increase, 2% of the vegetable market is involved in the production of RTE vegetables, reaching a turnover of about 600 million Euros [3,4]. 

The commercial success of these products is linked to the explicit and implicit services they offer: fresh, safe, healthy and nutritionally valuable products that can be consumed without preparation time are appealing to consumers who desire to improve their diet and save time [5]. Moreover, RTESs are 100% edible and are socially perceived as very high-quality products [6,7].

RTE food products are minimally processed products intended to be consumed without additional treatments. RTE leafy green vegetable processing includes several steps: after a first selection and elimination of external wilted or ruined leaves, the selected leaves are cut, washed, dried and packed in plastic containers [8,9]. The minimal technological processing ensures the preservation of organoleptic properties but is related to a generally shorter shelf-life compared to the starting product. The average shelf-life of RTESs ranges from 5 to 7 days, and, after packages have been opened, products can be stored at refrigeration temperatures lower than 8 °C for a maximum of 2 days. Modified atmosphere packaging (MAP) has been introduced as an upgrading technology to extend shelf-life and is currently adopted by major industries but is still not always implementable in small- and medium-sized enterprises (SMEs), which represent an important market sector in many countries, including Italy [10].

The main issue associated with these products is the high microbiological risk associated with their consumption. Microbiological contamination is common and inevitable in vegetables growing in soil. Typical environmental microorganisms found in soil and irrigation water contaminate plants infiltrating through roots or exposed (wounded or cut) surfaces and get internalized by the plant’s coating that creates a natural biofilm that protects them from surface treatments. The microflora can be further modified by other microorganisms that come in contact with the product during each step of the production chain [11,12,13,14,15,16]. 

RTESs are in fact involved in the transmission of foodborne pathogens: the high moisture content, the permissive pH (6.0–7.0), the lack of stringent decontamination procedures and the impact of temperature abuse during processing, transportation and storage can further increase the risk associated with these products. The number of gastroenteritis cases associated with RTE vegetable consumption has been increasing in recent years [17,18,19,20] and several outbreaks have been connected to the consumption of salads contaminated by *Salmonella* spp. [21], *Listeria monocytogenes* [22,23] and *Escherichia coli* O157: H7 [5,24]. Furthermore, RTESs may have an important role in the spread of bacteria of clinical interest carrying antibiotic resistance genes [25,26]. 

According to European Regulation (EC) No 1441/2007, the absence of *Salmonella* spp. and concentrations of *L. monocytogenes* lower than 100 colony forming units (CFU)/g are considered essential criteria to define the safety of RTESs placed on the market during their shelf-life. Although no mandatory microbiological criteria include the evaluation of total aerobic mesophilic count (TAMC) or *E. coli*, several guidelines include these parameters as indicators of the overall microbiological quality of RTE foods’ production processes [27,28]. High concentration levels could be an indicator of inadequate treatment, a shorter shelf-life and an overall higher microbiological risk. In particular, in Portuguese guidelines [29], satisfactory, acceptable and not acceptable levels of TAMC and *E. coli* are specified for RTE salads (Table 1).

In recent years, several research groups have studied the microbiological quality of RTESs highlighting high counts of total aerobic mesophilic count, coliforms, yeasts and molds but no presence of *Salmonella* spp. and *L. monocytogenes* [30,31,32,33,34,35,36,37,38,39,40]. Less attention has been given to the evolution of microflora during shelf-life and during home-refrigeration after package opening.

The aim of this work is to evaluate the microbiological quality and safety of RTESs sold in widespread supermarket chains in Lazio, Italy, on the packaging date, during shelf-life and during home-refrigeration. The study also aims to determine the differences between low-, medium- and high-cost RTESs and the impact of MAP technology in terms of the quality and safety of these products. *Salmonella* spp. and *L. monocytogenes* were chosen as indicators of RTES safety while TAMC and *E. coli* were chosen as indicators of RTES quality. Pathogen detection was performed following reference ISO methods as required by the EU Regulation while the TAMC was performed according to reference ISO and the alternative Micro Biological Survey (MBS) method. The MBS method is a colorimetric system for the easy detection and selective count of bacteria present in agro-food, in water and in environmental samples [41], developed, produced, and commercialized by MBS srl, 00131 Rome (Italy), a former spin-off company of Roma Tre University. The method, which has already demonstrated efficiency in carrying out microbiological analyses [42,43,44], and its accuracy and repeatability in comparison with the reference method for TAMCs, has been largely demonstrated in previous works [41,45].

## 2. Materials and Methods 

### 2.1. Evaluation of RTES Microbiological Quality and Safety during Shelf-Life

Samples: Two different varieties of RTESs were selected among the products commercially available in Italian supermarkets: baby romaine lettuce (BRL) and rocket salad (RS). Varieties were chosen from a low-cost (LC) brand, sold in a popular discount supermarket, a medium-cost (MC) store-brand, sold in a higher-priced supermarket, and a high-cost (HC) brand-name. A total of 6 production batches for each variety and each category were analyzed on the packaging date and on the expiry date (total batches = 36). Two bags from the same batch were purchased on the packaging date and transported in their primary package under refrigeration conditions (4 ± 1 °C) to the laboratory. One was immediately analyzed, and the other one was stored at 4 °C, opened and analyzed on the expiry date.

Sample preparation: Samples were prepared homogenizing 30 g in 275 mL of Buffered Peptone Water (BPW, Applichem, Darmsdadt, Germany) using a Stomacher 400, Seward, London, UK for 120 s at medium speed and serially diluted in the same diluent when needed. 

Evaluation of the TAMC using the pour plate method: Evaluation of the TAMC was performed according to UNI EN ISO 4833-1:2013. Samples were prepared as previously described. One milliliter of the selected dilutions was transferred into the Petri dishes in triplicate, then 15 to 17 mL of Plate Count Agar (PCA) medium (Applichem, Darmsdadt, Germany) at 45 °C was poured into each Petri dish. Plates were inverted and incubated at 30 °C for 72 h. Colonies in plates with 25 to 250 colonies were counted and viable counts in the test sample per gram were calculated as follows:

N= ∑C/[n_1_ + 0.1n_2_)] × *d* (1)

where:

N = number of colonies per ml or gram of sample.
∑C = sum of all of the colonies in all plates counted.
n_1_ = number of plates in the lower dilution counted.
n_2_ = number of plates in the next higher dilution counted.
*d* = dilution factor corresponding to the first dilution retained.

Evaluation of *E. coli* using the pour plate method: An evaluation of beta-glucuronidase-positive *E. coli* was performed according to UNI EN ISO 16649-2:2010. Samples were prepared as previously described. One millileter of the selected dilutions was transferred into the Petri dishes in triplicate, then 15 to 17 mL of Tryptone Bile-glucuronide (TBX) agar medium (Applichem, Darmsdadt, Germany) at 45 °C was poured into each Petri dish. Plates were inverted and incubated at 44 °C for 24 h. Colonies displaying the typical morphological characteristics (blue to blue-green) in plates containing 15–150 typical CFU and less than 300 total (typical and non-typical) CFU were counted and the number of CFU of β-glucuronidase-positive *E. coli* present in the test sample per gram were calculated as follows:

N= ∑*a*/[(n_1_ + 0.1n_2_)] × *d* (2)

where:

N = number of colonies per ml or gram of sample.
∑*a* = sum of the CFU counted on all the dishes retained from two successive dilutions, at least one of which contains a minimum 15 blue CFU.
n_1_ = number of plates in the lower dilution counted.
n_2_ = number of plates in the next higher dilution counted.
*d* = dilution factor corresponding to the first dilution retained.

Evaluation of the TAMC using the MBS method: The MBS method is a colorimetric system for the detection and quantification of bacteria in food and water samples. A TAMC using the MBS method was performed using MBS Total Viable Count (TVC) vials, containing the specific lyophilized growth medium for the detection and quantification of viable mesophilic aerobic bacteria.

To start the analysis, vials were rehydrated with 10 mL of sterile distilled water and paraffin oil and shaken until all the reagent was dissolved. Vials were inoculated with 1 mL of the sample homogenate and its serial dilutions, in parallel with the reference pour plate method. All analyses were performed in triplicate. Vials were incubated at 30 °C for 30 h. 

The vials’ medium color was periodically controlled with a thermostatic colorimeter that automatically detects the color change. A color change from blue to yellow of the reaction medium is indicative of a positive result; i.e., the presence of aerobic mesophilic bacteria [45]. The time for color change after inoculum varies according to the bacterial concentration. The time for color change was inversely related to the bacterial content of the analyzed sample: the higher the bacterial concentration, the less time required for color change. The persistence of the starting color indicates a negative result; that is, an absence of the microorganisms of interest. Regression lines were obtained plotting the time taken for the TVC vials to change color against the logarithm of the TAMC concentration obtained with the reference method. 

Detection of pathogens of interest: A recovery of the pathogens of interest according to Regulation (EC) No 1441/2007 was performed according to UNI EN ISO 11290-2:2017 and UNI EN ISO 6579-1: 2017, respectively, for the enumeration of *L. monocytogenes* and for the detection of *Salmonella* spp. Both analyses were performed on the same food homogenate.

For the enumeration of *L. monocytogenes,* the food homogenate was left 1 h at room temperature and then 1 mL was plated in 3 PALCAM agar plates in duplicate and incubated at 37 °C for up to 48 h. Gray-green colonies surrounded by dark brown to black halos were cultured in Brain Heart Infusion (BHI) broth overnight at 37 °C and the confirmation was performed using the qualitative immunoassay for the determination of *Listeria monocytogenes* (LISTERIA M. CARD, InterMedical, Villaricca, NA, Italy).

For the detection of *Salmonella* spp., the food homogenate was incubated at 37 °C for 24 h. After this pre-enrichment step, 1 mL and 100 µl of the pre-enrichment broth were transferred, respectively, in 10mL of Muller Kauffmann Tetrathionate Broth and Rappaport Vassiliadis broth and incubated, respectively, at 37 °C and 44 °C for 24 h. Next, 10 µL of the two selective broths were spread in duplicate on Xylose Lysine Deoxycholate (XLD) agar and Brilliant Green agar (BGA) plates and incubated at 37 °C for 24 h. Five colonies (or all if < 5 CFU) displaying the typical morphological *Salmonella* characteristics (pinkish red colonies on BGA and red colonies with black centers) were cultured in BHI broth overnight at 37 °C and the confirmation was performed using the qualitative immunoassay for the determination of *Salmonella* spp. (SALMONELLA Ag CARD, InterMedical, Villaricca, NA, Italy). Additionally, 5 non-suspected colonies underwent confirmation following the same procedure. 

### 2.2. Evaluation of RTES Microbiological Quality Simulating Home-Refrigeration after Package Opening

Samples: Three different varieties of RTESs were selected among the products commercially available in Italian supermarkets: BRL, RS and lamb’s lettuce (LL). Varieties were chosen from an LC brand and a HC top-selling brand. A total of 3 production batches for each variety and each category were selected (total number of batches = 18). The bags were purchased on the packaging date, transported, in their primary package and under refrigeration conditions (4 ± 1 °C), to the laboratory and analyzed on the packaging date. The opened bags were re-sealed and analyzed after a 2-day storage at 4 °C, as per the manufacturer’s indication, simulating home-refrigeration conditions.

Sample preparation: Samples were prepared homogenizing 30 g in 275 mL of Buffered Peptone Water (BPW) using a Stomacher 400, Seward, London, UK for 120 s at medium speed and serially diluted in the same diluent. 

Evaluation of the TAMC using the pour plate method: An evaluation of the TAMC according to ISO 4833-1:2013 was performed as previously described.

Evaluation of the TAMC using the MBS method: An evaluation of the TAMC according to the MBS method was performed as previously described.

Statistical analysis: A statistical analysis of variance (ANOVA) and covariance (ANCOVA) were performed using Past (Paleontological Statistics package for education and data analysis) version 3.12 for Windows.

## 3. Results

### 3.1. Evaluation of RTES Microbiological Quality and Safety during Shelf-Life

BRL and RS samples were selected among the products commercially available in Italian supermarkets. The microbiological quality and safety of RTESs was evaluated during shelf-life: the enumeration of *L. monocytogenes,* the detection of *Salmonella* spp., TAMC and evaluation of *E. coli* were performed on the packaging and expiry dates. Table 2 shows the results obtained for pathogen recovery: all batches were compliant with European standards for *L. monocytogenes*; conversely 67% of the analyzed batches resulted positive for *Salmonella* spp. resulting in a non-compliant status with regard to European regulations. All the *Salmonella* positive batches were found to be positive both on the packaging and the expiry date. 

**Figure 1:**
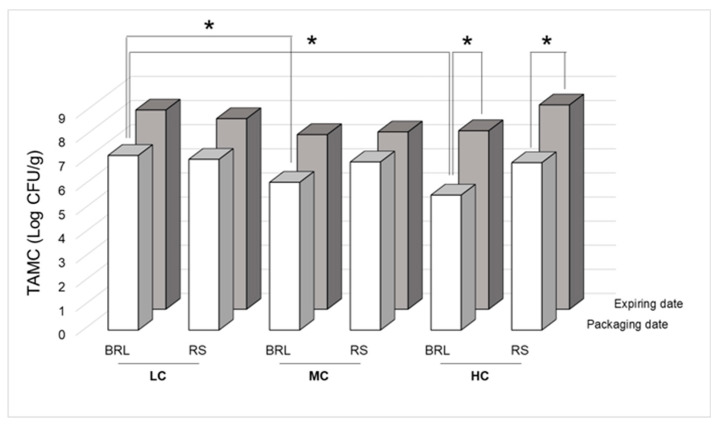
Ready-to-eat leafy green salads’ average total aerobic mesophilic count (TAMC) contamination levels at packaging and expiry date for low-cost (LC), medium-cost (MC) and high-cost (HC) baby romaine lettuce (BRL) and rocket salad (RS) samples (SD < 10%) evaluated using the plate count method. * Significant difference (*p* < 0.05).

TAMC results for BRL and RS are displayed in Figure 1. Of all the samples analyzed only 17% displayed an acceptable level of contamination according to Portuguese guidelines (>10^4^ ≤ 10^6^ CFU/g). 

The average concentration on the packaging date was of 6.63 (±0.64) and 7.63 (±0.42) log CFU/g, respectively, with an average growth of 1 log unit (+15%) on the expiry date. On the packaging date, 100%, 67% and 33% samples of BRL samples displayed unsatisfactory results (TAMC > 10^6^ CFU/g) for LC, MC and HC samples, respectively. For RS, on the packaging date, 100% of LC and MC and 83% of HC samples were unsatisfactory (TAMC > 10^6^ CFU/g). On the expiry date, all samples, independent of the variety and the cost category, displayed unsatisfactory results. A significant difference in the TAMC on the packaging date for BRL samples was observed between LC and MC and between LC and HC samples; no significant difference was observed for RS samples. A significant difference between the TAMC on the packaging date and expiry date was observed only for HC samples both for BRL and RS. The overall percent increase in growth for each variety and each cost category is displayed in Table 3.

All samples were acceptable regarding the presence of *E. coli* that was recovered in only one batch of MC rocket salad in concentration <100 CFU/g.

### 3.2. Evaluation of RTES Microbiological Quality Simulating Home-Refrigeration after Package Opening

**Figure 2:**
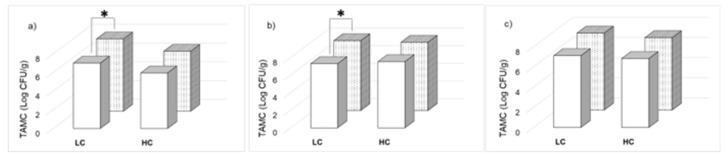
Ready-to-eat salad average total aerobic mesophilic count (TAMC) contamination levels at packaging date (□) and after 2 days of storage of the opened bags at 4 °C (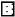) for low-cost (LC) and high-cost (HC) samples: (**a**) baby romaine lettuce (BRL); (**b**) rocket salad (RS); (**c**) lamb’s lettuce (LL) samples (SD < 10%) evaluated using the plate count method * Significant difference (*p* < 0.05).

Baby romaine lettuce, rocket salad and lamb’s lettuce samples were selected among the products commercially available in Italian supermarkets. The percent increase in growth after package opening and simulating home-refrigeration was evaluated on the packaging date and after 2 days of storage of the open bags at 4 °C. The TAMC results for BRL, RS and LL are displayed in Figure 2 (Figure 2a–c respectively). On the packaging date, 100% and 33% of the samples of the analyzed batches displayed unsatisfactory results (TAMC > 10^6^ CFU/g) for LC and HC samples, respectively. The average concentrations on the packaging and expiry date were 6.96 (±0.55) and 7.45 (±0.56) log CFU/g, respectively, with an overall growth of 0.5 log unit (+7%). 

Two days after opening, all the analyzed samples displayed a TAMC concentration >10^6^ CFU/g. A significant difference between TAMC on the packaging date and after 2 days of storage of the opened bags at 4 °C was observed only for LC, BRL and RS samples; no significant difference was observed for HC samples independently from the variety. The overall percent increase in growth for each variety and each cost category is displayed in Table 4.

### 3.3. Accuracy of the MBS Method

**Figure 3:**
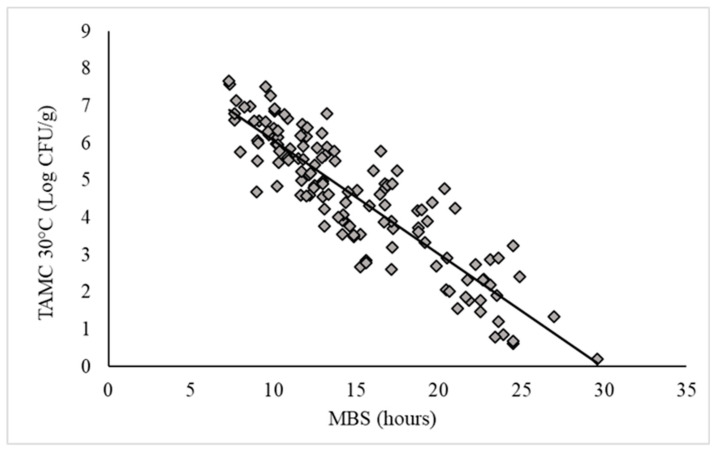
Correlation line between the results obtained with the Micro Biological Survey (MBS) method and reference methods. The total aerobic mesophilic count (TAMC) quantitative results obtained with reference method were plotted against the time taken for the MBS total viable count vials to change color. Continuous line represents the linear regression analysis (slope = 0.30, R^2^ = 0.79). Each analysis was performed in triplicate (SD < 0.4).

TAMC analyses were performed with the reference method and the alternative MBS method. The linearity of the MBS method was evaluated according to ISO 16140:2016. A correlation between the time taken for the MBS TVC vials to change color and the log CFU/mL of TAMC is shown in Figure 3. A linear inverse relationship between the time for color change of the MBS TVC vials and the TAMC at 30 °C (log CFU/g) was observed (slope = 0.30; maximum analysis time= 30 h; R^2^ = 0.79) (Figure 3).

In this work, we have analyzed the microbiological quality and safety of RTES sold in Lazio, Italy, taking into consideration different varieties and cost categories.

With regard to pathogens, interestingly, the prevalence of *Salmonella* spp. was very high (67%) and no significant difference could be observed among the selected varieties and cost categories; *L. monocytogenes* was not recovered, aligning with the results obtained in other studies [34,35,36,37,38]. The detection of *Salmonella* spp. was performed according to UNI EN ISO 6579-1:2017, as required by the European Regulation. The divergence between the results obtained in this work from those obtained by other groups regarding the presence of *Salmonella* spp. could be explained by the fact that most of the positive results following immunological confirmation were obtained from non-suspected colonies, displaying non-typical morphological characteristics.

The high recovery rate of microorganisms belonging to the genus *Salmonella* in the analyzed RTES samples, independent of varieties and cost categories, is worthy of note and must be further investigated. The European Regulation requires the absence of *Salmonella* spp. regardless of species identification: the positive samples found in this study are not compliant with the abovementioned criteria and should not have been marketed, even though the effective impact on consumer health cannot be assessed. However, the absence of Salmonella spp. in ready-to-eat products is a mandatory safety criterion in all the European Union Member States and all products placed on the European market must be compliant with this criterion. Moreover, many high-cost brands that are marketed in Italy also produce and sell their products in other European countries and all over the world, making the results of this study significant for the fresh-food industry operating in the ready-to-eat salad sector.

The study of TAMC during shelf-life revealed many unsatisfactory results: on the packaging date, HC batches were less contaminated compared to LC and MC samples, probably linked to the specific packaging conditions (MAP). On the expiry date, all batches displayed unsatisfactory results: an average higher percent increase in growth has been observed in HC salads compared to LC and MC salads. We hypothesize that this could be due to the fact that the MAP condition could affect the existing microflora by reducing the initial contamination level and selecting a specific microflora that, in the unaltered MAP product environment, benefits from a higher availability of nutrients and reduced competition compared to the the microflora found in LC and MC samples. In these products, given that the environmental conditions have not been modified, and a high bacterial load is already present on the packaging date, the overall growth of resident microflora is limited by competition among the microorganisms and, in general, the carrying capacity of the specific environment. A slightly different trend was observed when simulating home-refrigeration after package opening. On the packaging date, HC batches were less contaminated compared to LC baby romaine lettuce while no significant difference was observed for the other samples. After two days of storage at 4 °C of the opened bags, all the analyzed samples displayed unsatisfactory results; the growth trends were similar to those observed during shelf-life for LC batches while they were considerably reduced for HC batches. This difference may be indicative of the fact that the altered MAP environmental condition caused by the bags being opened could in some way affect the metabolism of resident microflora.

A TAMC were performed in parallel with the MBS method with satisfying outcomes. The MBS method was quite accurate (R^2^ = 0.78) and was able to detect <5 CFU/mL in 30 h, significantly reducing the standard analytical times (72 h). The simple procedure, the simplified interpretation of results and the stand-alone equipment could be very useful to streamline microbiological analyses, particularly in small and medium enterprises. 

## 4. Conclusions

The microbiological quality and safety of RTESs still seems to be a challenge despite the advances in technology and the attention from Regulations and International food safety agencies. The obtained results highlight the need for more extensive microbiological control and suggest the optimization of large-scale washing procedures. A more attentive analysis of the possible conditions occurring during shelf-life and domestic storage should also be considered. The implementation of an accurate, fast, easy and portable microbiological method of analysis could be a valuable tool to provide higher quality products. 

## Figures and Tables

**Table 1:** Satisfactory, acceptable and not acceptable total aerobic mesophilic count (TAMC) and *E. coli* contamination levels (colony forming units per gram) for ready-to-eat leafy green salads according to Portuguese guidelines [29].

Indicators	Contamination Levels (CFU/g)		
	Satisfactory	Acceptable	Not Acceptable
TAMC	≤10^4^	>10^4^ ≤ 10^6^	>10^6^
*E. coli*	≤10	>10 ≤ 10^2^	>10^2^

**Table 2:** Ready-to-eat leafy green salad batches acceptability according to European Regulation (EC) No 1441/2007 safety criteria: for *Salmonella* spp. absence in 25 g, for *L. monocytogenes* lower than 100 colony forming units (CFU)/g.

	*Salmonella* spp.	*L. monocytogenes*
Compliant	12	36
Non-compliant	24	0
Total	36	36

**Table 3:** Ready-to-eat leafy green salad average total aerobic mesophilic count (TAMC) percent increase in growth from packaging to expiry date for each variety and cost category.

	LC	MC	HC
BRL	+11%	+18%	+29%
RS	+10%	+5%	+20%
Overall	+10.5%	+11.5	+24.5%

**Table 4:** Ready-to-eat leafy green salad average total aerobic mesophilic count (TAMC) percent increase in growth after package opening during a simulated home-refrigeration of 2 days at 4 °C for each variety and cost category.

	LC	HC
Baby romaine lettuce	+11%	+8%
Rocket salad	+9%	+3%
Lamb’s lettuce	+7%	+5%
Overall	+9%	+5.3%
